# Genetic and environmental contributions to diagnostic fluctuation in anorexia nervosa and bulimia nervosa

**DOI:** 10.1017/S0033291719002976

**Published:** 2021-01

**Authors:** Shuyang Yao, Henrik Larsson, Claes Norring, Andreas Birgegård, Paul Lichtenstein, Brian M. DʼOnofrio, Catarina Almqvist, Laura M. Thornton, Cynthia M. Bulik, Ralf Kuja-Halkola

**Affiliations:** 1 Department of Medical Epidemiology and Biostatistics, Karolinska Institutet, Stockholm, Sweden; 2 School of Medical Sciences, Örebro University, Örebro, Sweden; 3 Department of Clinical Neuroscience, Centre for Psychiatry Research, Stockholm, Karolinska Institutet, & Stockholm Health Care Services, Stockholm County Council, Sweden; 4 Department of Psychological and Brain Sciences, Indiana University, Bloomington, USA; 5 Astrid Lindgren Children's Hospital, Karolinska University Hospital, Stockholm, Sweden; 6 Department of Psychiatry, University of North Carolina at Chapel Hill, Chapel Hill, North Carolina, USA; 7 Department of Nutrition, University of North Carolina at Chapel Hill, Chapel Hill, North Carolina, USA

**Keywords:** Anorexia nervosa, behavioural genetics, bulimia nervosa, clinical diagnosis, eating disorders, genetic epidemiology, medical register, sibling design

## Abstract

**Background:**

Anorexia nervosa and bulimia nervosa are two severe eating disorders associated with high premature mortality, suicidal risk and serious medical complications. Transition between anorexia nervosa and bulimia nervosa over the illness course and familial co-aggregation of the two eating disorders imply aetiological overlap. However, genetic and environmental liabilities to the overlap are poorly understood. Quantitative genetic research using clinical diagnosis is needed.

**Methods:**

We acquired a clinical diagnosis of anorexia nervosa (prevalence = 0.90%) and bulimia nervosa (prevalence = 0.48%) in a large population-based sample (*N* = 782 938) of randomly selected full-sisters and maternal half-sisters born in Sweden between 1970 and 2005. Structural equation modelling was applied to quantify heritability of clinically diagnosed anorexia nervosa and bulimia nervosa and the contributions of genetic and environmental effects on their overlap.

**Results:**

The heritability of clinically diagnosed anorexia nervosa and bulimia nervosa was estimated at 43% [95% confidence interval (CI) (36–50%)] and 41% (31–52%), respectively, in the study population, with the remaining variance explained by variance in unique environmental effects. We found statistically significant genetic [0.66, 95% CI (0.49–0.82)] and unique environmental correlations [0.55 (0.43–0.66)] between the two clinically diagnosed eating disorders; and their overlap was about equally explained by genetic and unique environmental effects [co-heritability 47% (35–58%)].

**Conclusions:**

Our study supports shared mechanisms for anorexia nervosa and bulimia nervosa and extends the literature from self-reported behavioural measures to clinical diagnosis. The findings encourage future molecular genetic research on both eating disorders and emphasize clinical vigilance for symptom fluctuation between them.

## Introduction

Eating disorders are psychiatric illnesses marked by dysregulated appetite, eating behaviours, and shape and weight concerns. Anorexia nervosa is characterized by significantly low body weight and intense fear of weight gain (Dalle Grave *et al*., 2013) and affects approximately 1% females and 0.3% males (Hudson *et al*., 2007; Preti *et al*., 2009; Thomas *et al*., 2016) worldwide. Bulimia nervosa, affecting approximately 1.5% females and 0.5% males (Hudson *et al*., 2007; Preti *et al*., 2009; Thomas *et al*., 2016), is characterized by recurrent episodes of uncontrollable binge eating coupled with compensatory behaviours (e.g. purging, fasting and excessive exercise) to prevent consequent weight gain (Dalle Grave *et al*., 2013). Both eating disorders have been associated with increased premature mortality and suicidal risk (Arcelus *et al*., 2011; Whiteford *et al*., 2013; Keshaviah *et al*., 2014; Yao *et al*., 2016) and medical (Kessler *et al*., 2013; Sheehan and Herman, 2015; Westmoreland *et al*., 2016) and psychiatric comorbidities (Herzog *et al*., 1992; Striegel-Moore *et al*., 1999; Kaye *et al*., 2004; Keel *et al*., 2005; Hudson *et al*., 2007; Bulik-Sullivan *et al*., 2015; Cederlof *et al*., 2015).

Although anorexia nervosa and bulimia nervosa are defined as two distinct eating disorders, they share common symptoms. For instance, both disorders include over-evaluation of body weight and shape and behaviours to control weight; anorexia nervosa can include binge eating and purging behaviours, and bulimia nervosa can also involve restrictive eating behaviours. Diagnostic crossover has also been observed across the course of illness. Although pure forms of the illnesses exist, 10–54% of individuals with anorexia nervosa develop bulimia nervosa and 2–27% of individuals with bulimia nervosa develop anorexia nervosa during the course of illness (Tozzi *et al*., 2005; Eddy *et al*., 2008; Peat *et al*., 2009; Fichter *et al*., 2017). The two eating disorders also share common comorbid conditions such as mood disorders, anxiety disorder, substance use disorders and attention-deficit hyperactivity disorder (Hudson *et al*., 2007; Yao *et al*., 2019) In terms of treatment, transdiagnostic approaches such as enhanced cognitive behavioural therapy are associated with favourable outcomes in both anorexia nervosa and bulimia nervosa (Dalle Grave *et al*., 2013; Fairburn *et al*., 2013; Fairburn *et al*., 2015). Together, these observations suggest considerable, but not complete, overlap between anorexia nervosa and bulimia nervosa.

Observed familial co-aggregation of the two eating disorders further implies their aetiological overlap; relatives of individuals with anorexia nervosa have increased risk (over four times) of bulimia nervosa compared to the same types of relatives of individuals without anorexia nervosa, and *vice versa* (Stein *et al*., 1999; Strober *et al*., 2000). To our knowledge, only one study has quantified the genetic and environmental effects on the overlap between anorexia nervosa and bulimia nervosa (Bulik *et al*., 2010). Using self-ratings of eating behaviours in a Swedish twin sample, the study reported high genetic [0.78, 95% confidence interval (CI) (0.51–1.00)] and moderate unique environmental [0.44 (0.20–0.65)] correlations between anorexia nervosa and bulimia nervosa. However, due to the relatively low prevalence of both disorders and of twinning, clinical diagnoses have rarely been used in quantitative genetic studies of eating disorders.

In the current work, we used clinical diagnoses of anorexia nervosa and bulimia nervosa in a large Swedish female cohort containing full-sisters (sisters born to the same biological parents) and maternal half-sisters (sisters born to the same biological mother but different biological fathers). The application of diagnostic information considerably expands the clinical significance of the results. Using non-twin siblings significantly increased sample size and improved precision in estimating the underlying morbidity liabilities. We quantified the contribution of genetic and environmental effects on anorexia nervosa and bulimia nervosa and their overlap and estimated the genetic and environmental correlations between the two eating disorders.

## Methods and materials

The current study was approved by the Regional Ethics Review Board in Stockholm, Sweden.

### Register data

We acquired information from several Swedish national registers linked by the unique individual identification number. The Swedish Population Register provided birth year and month, death date and migration information (Ludvigsson *et al*., 2016). The Multi-Generation Register contained information on biological parents for each individual who was born after 1932 and lived in Sweden since 1961. The National Patient Register (NPR) included inpatient psychiatric diagnoses since 1973 and outpatient psychiatric diagnoses since 2001; diagnoses of eating disorders in the NPR were based on the Swedish versions of the International Classification of Diseases, Ninth Revision (ICD-9 1987–1996) and Tenth Revision (ICD-10, since 1997) (Ludvigsson *et al*., 2011). The Swedish National Quality Register for Eating Disorder Treatment (Riksät, since 1999) and quality assurance system for eating disorders (Stepwise, since 2005) were two treatment quality registers for eating disorder treatments from specialized centres across Sweden (Birgegard *et al*., 2010; Emilsson *et al*., 2015; Javaras *et al*., 2015); diagnoses in the treatment quality registers were based on the Diagnostic and Statistical Manual of Mental Disorders, Fourth Edition, Text Revision (DSM-IV-TR). Data from the registers were updated until 2013–12–31.

Anorexia nervosa diagnosis was identified with ICD-9 code 307B or ICD-10 codes F50.0 or F50.1 from the NPR, or meeting DSM-IV-TR criteria for anorexia nervosa or sub-threshold anorexia nervosa in Eating Disorder Not Otherwise Specified (EDNOS) from the treatment quality registers. We were unable to distinguish between restricting and binge-eating and purging anorexia nervosa from the ICD diagnosis in the NPR. Bulimia nervosa diagnosis was identified with ICD-10 codes F50.2 or F50.3 from the NPR, or meeting DSM-IV-TR criteria for bulimia nervosa or sub-threshold bulimia nervosa in EDNOS from the treatment quality registers. Bulimia nervosa was not an independent eating disorder category in the Swedish version of ICD-9 (1987). Therefore, its diagnostic period was shorter than that of anorexia nervosa, which could lead to under-detection of bulimia nervosa cases relative to anorexia nervosa cases.

### Study population

Through the Swedish Population Register and the Multi-Generation Register, we identified all full-sisters (sisters born to the same parents) and maternal half-sisters (sisters born to the same mother but different fathers) who were born in Sweden between 1970 and 2005. We excluded individuals who emigrated or died before age 6, adopted individuals and individuals whose biological parents were not identifiable from the registers. We did not have access to information on race and ethnicity; however, being born in Sweden might suggest that our study population was comprised primarily of individuals of Scandinavian/Nordic ancestry. We then randomly selected one pair of maternal half-sisters per mother [57 036 pairs, mean age 25.2 years by the end of follow-up in year 2013, standard deviation (s.d.) = 9.3]. We then randomly selected one pair of full-sisters per mother in the remaining mothers after excluding mothers of the selected maternal half-sisters. We excluded twin pairs from full-sister pairs, resulting in 334 433 pairs of full-sisters for analysis (mean age 25.5 years by year 2013, s.d. = 9.6). No individual was included in more than one pair. The selected full-sisters and maternal half-sisters comprised the study population, where 6104 (0.91%) individuals among the full-sisters and 938 (0.82%) among the maternal half-sisters had been diagnosed with anorexia nervosa; 3142 (0.47%) among the full-sisters and 579 (0.51%) among the maternal half-sisters had been diagnosed with bulimia nervosa; 679 (0.10%) among the full-sisters and 122 (0.11%) among the half-sisters had been diagnosed with both disorders (Table 1).

**Table 1.** Descriptive statistics and correlations

	Full-sisters	Maternal half-sisters
Total *N*	668 866	114 072
Within disorder		
Anorexia nervosa		
*n* (%)	6104 (0.91%)	938 (0.82%)
Age at first diagnosis, range	7.4–42.1	7.6–41.7
Age at first diagnosis, mean (s.d.)	18.6 (4.9)	19.1 (5.6)
Con./Dis. Pair^a^	107/5890	5/928
Cross-sister-within-trait correlation	0.22 (0.02)	0.04 (0.06)
Adjusted cross-sister-within-trait correlation	0.22 (0.02)	0.03 (0.06)
Bulimia nervosa		
*n* (%)	3142 (0.47%)	579 (0.51%)
Age at first diagnosis, range	8.9–41.4	13.6–41.4
Age at first diagnosis, mean (s.d.)	23.2 (5.2)	23.5 (5.7)
Con./Dis. Pair^a^	34/3074	4/571
Cross-sister-within-trait correlation	0.21 (0.03)	0.13 (0.07)
Adjusted cross-sister-within-trait correlation	0.20 (0.03)	0.13 (0.07)
Cross disorder		
Within individual		
*n* (%)^b^	679 (0.10%)	122 (0.11%)
Phenotypic correlation	0.59 (0.01)	0.60 (0.02)
Adjusted phenotypic correlation	0.59 (0.01)	0.60 (0.02)
Cross sister		
Con./Dis. Pair^c^	76/9094	6/1505
Cross-sister-cross-trait correlation	0.14 (0.02)	0.03 (0.05)
Adjusted cross-sister-cross-trait correlation	0.14 (0.02)	0.03 (0.06)

The presented correlations are tetrachoric correlations (with standard error). Phenotypic correlation was the correlation of anorexia nervosa and bulimia nervosa within an individual; cross-sister-within-trait correlation was the correlation of one disorder between the two sisters in a pair; cross-sister-cross-trait correlation was the correlation between anorexia nervosa in one sister and bulimia nervosa in the other sister in a pair. We presented both the crude correlations and the correlations adjusted for birth year.

a A concordant pair was a pair where both sisters had the disorder; a discordant pair was a pair where only one of the sisters had the disorder.

b The number (and prevalence) of individuals with both anorexia nervosa and bulimia nervosa.

c Here a concordant pair was a pair where one sister had anorexia nervosa and the other had bulimia nervosa, and a discordant pair was a pair where one sister had anorexia or bulimia nervosa, and the other sister did not have the other disorder.

### Statistical analysis

We first examined the distribution of anorexia nervosa and bulimia nervosa and their correlations in full-sisters and maternal half-sisters. We estimated phenotypic correlations (i.e. correlation between anorexia nervosa and bulimia nervosa within an individual), cross-sister-within-trait correlations (i.e. correlation of one disorder between the two individuals in a pair) and cross-sister-cross-trait correlations (i.e. correlation between anorexia nervosa in one individual and bulimia nervosa in the other individual in the pair). The correlations were tetrachoric correlations in this study as the diagnoses were binary variables (whether the individual received the diagnosis or not). The estimates were obtained with a liability-threshold model where normally distributed underlying liabilities to the binary phenotypes were assumed (Neale and Cardon, 1992; Bulik *et al*., 2000). We adjusted for birth year of the individuals.

We then applied structural equation modelling (Neale and Cardon, 1992) to decompose the aforementioned correlations and estimate the genetic and environmental effects on anorexia nervosa and bulimia nervosa and their overlap. Specifically, four effects were estimated in the study. Additive genetic effects (A) represented the cumulative influence of multiple genes, each of which was presumed to have a small effect on the phenotype. Dominance genetic effects (D) were modelled to measure the genetic effects deviated from the additive effects. Shared environmental effects (C) were defined as environmental impact on both individuals in a pair (i.e. which made the two sisters similar to each other), whereas unique environmental effects (E, including measurement error) influence one individual but not the other in a pair (Plomin *et al*., 2013). Full-sisters share 50% of their segregating alleles on average and therefore 50% additive genetic effects and 25% dominance genetic effects; whereas maternal half-sisters share 25% of additive genetic effects on average and no dominance genetic effects. As most maternal half-siblings live in the same family just as full-siblings in our study population (1994), the two types of sisters were assumed to have equal shared environmental effects. Structural equation models quantified the proportions of phenotypic variation attributable to all or some of the A, D, C and E components.

**Fig. 1.**
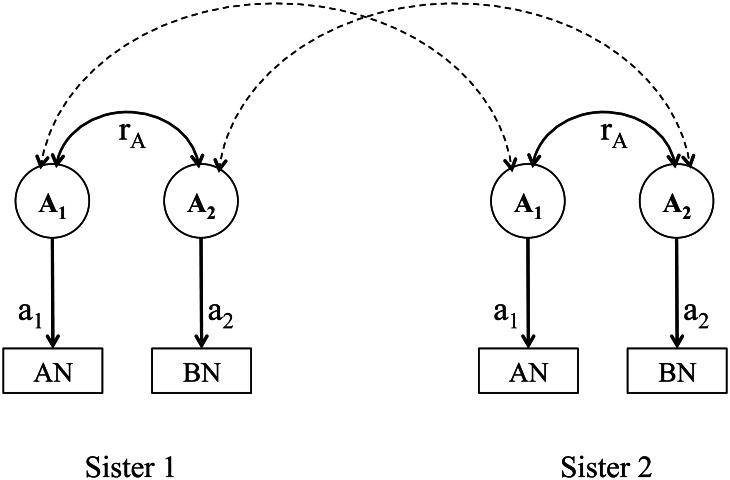
Path diagram of the additive genetic effects and genetic correlations in a bivariate model of anorexia nervosa and bulimia nervosa. AN, anorexia nervosa; BN, bulimia nervosa. *A*_1_ represents the latent additive genetic effect that contributes a_1_ to anorexia nervosa; *A*_2_ represents the latent additive genetic effect that contributes a_2_ to bulimia nervosa. The additive genetic correlation between anorexia nervosa and bulimia nervosa is represented by *r*_A_. Parameters a_1_, a_2_ and *r*_A_ are the unknown parameters. The dashed double arrows indicate the genetic sharing between two sisters in a pair; they are 0.5 for full-sisters and 0.25 for maternal half-sisters.

Given the data of two types of siblings, at most three of the four components could be estimated at a time. We fitted three bivariate structural equation models, namely models including components A, C and E (an ACE model), A, D and E (an ADE model), and A and E (an AE model), using the OpenMx package (version 2.8.3) (Neale *et al*., 2016) in R (version 3.3.3). Weighted least squares method was used for model fitting and estimating the standard errors. Scripts are available from the corresponding author upon reasonable request. Figure 1 illustrates the path diagram of the A component in the models; other components included in the models follow the same logic but are not shown in the figure for simplicity.

We detected that the prevalence of the disorders differed slightly between full-sisters and maternal half-sisters (χ^2^ test, *p* = 0.003 for anorexia nervosa, and *p* = 0.090 for bulimia nervosa), and therefore allowed the thresholds to be freely estimated across relative types in all models. Likelihood ratio test was performed for each bivariate model against the saturated model to evaluate whether the bivariate model fitted the data similarly well compared to the saturated model (*p* > 0.05 indicates that the fittings were similarly well). We interpreted the results of the model with the lowest Akaike Information Criterion (AIC), which comprehensively reflects the goodness of fit (also reflected by likelihood ratio test) and model parsimony.

## Results

### Genetic and environmental effects on clinically diagnosed anorexia nervosa and bulimia nervosa

In the current study, we included clinical diagnostic information from 334 433 pairs of full-sisters and 57 036 pairs of maternal half-sisters. The cross-sister-within-trait correlation for anorexia nervosa was estimated as 0.22 [standard error (s.e.) = 0.02] in full-sisters and 0.03 (s.e. = 0.06) in maternal half-sisters; the cross-sister-within-trait correlation for bulimia nervosa was estimated as 0.20 (s.e. = 0.03) in full-sisters and 0.13 (s.e. = 0.07) in maternal half-sisters (Table 1). The higher resemblance in full-sisters than in maternal half-sisters indicates that genetic factors contribute to the liability to the eating disorders, because full-sisters on average share more segregating genes than maternal half-sisters, whereas the environmental contribution within a pair was assumed to be equal between the two types of sisters.

The models fitted the data similarly well compared to the saturated model (*p* values >0.05 in likelihood ratio tests against the saturated model). The AE model was selected for interpretation because it had the lowest AIC (Table 2). The model revealed moderate genetic and unique environmental effects on both anorexia nervosa and bulimia nervosa. The heritability of anorexia nervosa was estimated as 43% [95% CI (36–50%)], suggesting that 43% of the variance of anorexia nervosa in the population was attributable to the genetic variance in the population and the remaining variance [57% (50–64%)] was attributable to unique environmental variance. Approximately 41% (31–52%) of the observed variance of bulimia nervosa was explained by genetic variance and the remaining variance [59% (48–70%)] was attributable to unique environmental variance (AE model in Table 3).

**Table 2.** Model fitting statistics

	Number of estimated parameters	AIC	*p* value (against saturated model)^a^
Saturated model	29	303.13	NA
ACE model	20	295.02	0.31
ADE model	20	295.02	0.24
AE model	17	**291.61**	0.41

ACE model included components of additive genetic effects (A), shared environmental effects (C) and unique environmental effects (E). ADE model included components of A, dominant genetic effects (D) and E. AE model included components of A and E.

a The goodness of fit of ACE, ADE and AE models were similar to that of the saturated model (*p* values >0.05 in likelihood ratio tests against the saturated model). AE model had the lowest AIC, suggesting it was more parsimonious than ACE and ADE models. We therefore selected the estimates from AE model as the main results for interpretation.

**Table 3.** Genetic and environmental contributions to anorexia nervosa, bulimia nervosa, and their overlap, and the genetic and environmental correlations between the two eating disorders estimated from bivariate ACE, ADE and AE models (with 95% CI)

	A	D	C	E
Anorexia nervosa				
ACE	43% (0–93%)	NA	0 (0–25%)	57% (31–84%)
ADE	5% (0–54%)	77% (0–100%)	NA	18% (0–69%)
**AE**	**43% (36–50%)**	**NA**	**NA**	**57% (50–64%)**
Bulimia nervosa				
ACE	21% (0–80%)	NA	10% (−19 to 38%)	70% (37–100%)
ADE	35% (0–91%)	11% (0–100%)	NA	54% (0–100%)
**AE**	**41% (31–52%)**	**NA**	**NA**	**59% (48–70%)**
Overlap				
ACE	50% (−35 to 135%)	NA	−2% (−43 to 40%)	52% (7–96%)
ADE	22% (−59 to 104%)	49% (−115 to 214%)	NA	28% (−56 to 113%)
**AE**	**47% (35–58%)**	**NA**	**NA**	**54% (42–65%)**
	*r*_A_	*r*_D_	*r*_C_	*r*_E_
Correlation				
ACE	1.00 (−0.63 to 1.00)	NA	−1.00 (−1.00 to 1.00)	0.49 (0.12–0.85)
ADE	1.00 (−1.00 to 1.00)	1.00 (−1.00 to 1.00)	NA	0.54 (−0.93 to 1.00)
**AE**	**0.66 (0.49–0.82)**	**NA**	**NA**	**0.55 (0.43–0.66)**

**A** stands for additive genetic effects; **C** stands for shared environmental effects; **D** stands for dominant genetic effects; **E** stands for unique environmental effects. The effect of each on anorexia nervosa, bulimia nervosa and their overlap was estimated, adjusted for birth year. We primarily interpret results from AE model (with the lowest AIC).

### Genetic and environmental effects on the overlap between anorexia nervosa and bulimia nervosa

We observed statistically significant phenotypic correlations between clinically diagnosed anorexia nervosa and bulimia nervosa for both types of sisters (0.59, s.e. = 0.01 for full-sisters and 0.60, s.e. = 0.02 for maternal half-sisters), illustrating the diagnostic transition between the two eating disorders within individuals regardless of the type of siblings. The cross-sister-cross-trait correlation further characterizes the nature of the association between the disorders across the individuals in a pair. A higher cross-sister-cross-trait correlation was observed in full-sister pairs (0.14, s.e. = 0.02) than in maternal half-sisters (0.03, s.e. = 0.06), suggesting genetic effects on the overlap between the two eating disorders (Table 1).

The contribution of genetic effect to the overlap between anorexia nervosa and bulimia nervosa was measured by the proportion of the correlation of the two disorders explained by the covariance of the genetic effect on anorexia nervosa and the genetic effect on bulimia nervosa. The contribution of the unique environmental effect on the overlap was defined analogously. The overlap between anorexia nervosa and bulimia nervosa was about equally explained by genetic and unique environmental factors: approximately 46% [95% CI (35–58%)] of the phenotypic correlation of the two disorders was explained by their genetic covariance and the remaining 54% [95% CI (42–65%)] was explained by their environmental covariance (Table 3). We found significant genetic correlation [0.66, 95% CI (0.49–0.82)] and environmental correlation [0.55, 95% CI (0.43–0.66); Table 3] between anorexia nervosa and bulimia nervosa.

## Discussion

In this study, we quantified the genetic and environmental effects on clinically diagnosed anorexia nervosa and bulimia nervosa and their overlap. The findings of significant genetic [0.66, 95% CI (0.49–0.82)] and environmental correlations [0.55 (0.43–0.66)] suggest an aetiological overlap between anorexia nervosa and bulimia nervosa and underscore the importance of vigilance for transitions between the two eating disorders during treatment. Using large population data, we significantly improved the precision of the estimates. We reported heritability for clinically diagnosed anorexia nervosa as 43% (36–50%) and bulimia nervosa as 41% (31–52%), which was slightly lower than, but within the confidence interval of, the heritability found in the twin study based on self-reported questionnaire data [57% (0–81%) for anorexia nervosa and 62% (8–70%) for bulimia nervosa] (Bulik *et al*., 2010). The single-nucleotide polymorphism (SNP)-based heritability for anorexia nervosa has been estimated between 11% and 17% (s.e. 1%) in the most updated genome-wide association study (GWAS) (Watson and Yilmaz, 2019), which appeared lower than the heritability estimated in quantitative genetic studies. SNP-based heritability is not yet available for bulimia nervosa, as currently there is no GWAS on bulimia nervosa. The discrepancy between SNP-based heritability and heritability estimated from twin and sibling models has been observed for various phenotypes (Manolio *et al*., 2009). One possible explanation is that SNP-based heritability did not capture the heritability from alleles whose effects were too small to pass stringent significant tests (Yang *et al*., 2010).

Our finding of significant genetic correlation between clinically diagnosed anorexia nervosa and bulimia nervosa is in line with the previous twin study based on self-reported disordered eating conditions (Bulik *et al*., 2010). The significant genetic and environmental correlations found in both studies may reflect nosological overlap, but may also be explained by biological pleiotropic effects on both disorders and even other explanations, such as a causal influence of one disorder on the other (Martin *et al*., 2018). Nevertheless, there is no clear hypothesis on a directed causal effect between the two disorders, as transmission between anorexia nervosa and bulimia nervosa has been observed in both directions in clinical settings; approximately 10–54% patients with anorexia nervosa were later diagnosed with bulimia nervosa and approximately 2–27% with bulimia nervosa were later diagnosed with anorexia nervosa (Peat *et al*., 2009). It was also difficult to determine the temporal order of the onset of the two disorders based on registered diagnosis in the treatment-seeking sample, as the date of diagnosis was not necessarily the date of onset. Therefore, we did not attempt to discern the directionality of phenotypic association. Studies with accurate measures of the temporal order of the disorders (e.g. age at onset of the eating disorders) and pathological questions may be able to elucidate the developmental process underlying a directed association between anorexia nervosa and bulimia nervosa.

The observed genetic correlation between the two eating disorders was higher than that found between eating disorders and other psychiatric disorders. The highest genetic correlations were found between anorexia nervosa and obsessive-compulsive disorder [0.52 (0.26–0.81)] and between anorexia nervosa and major depression (around 0.5 in multiple studies) (Wade *et al*., 2000; Cederlof *et al*., 2015; Thornton *et al*., 2016), which were comparable but lower than the genetic correlation we estimated between the two eating disorders [0.66, 95% CI (0.49–0.82)]. SNP-based genetic correlation between anorexia nervosa and schizophrenia was lower [0.19 (0.11–0.27)] (Bulik-Sullivan *et al*., 2015), as well as the genetic correlations with attention-deficit hyperactivity disorder in the same study population [0.14 (0.05–0.22) for anorexia nervosa and 0.37 (0.31–0.42) for bulimia nervosa] (Yao *et al*., 2019). Such differences might reflect greater degrees of pleiotropy between the two eating disorders than between eating disorders and other psychiatric disorders.

In addition, we reported significant unique environmental effects, both disorder-specific and common to the two eating disorders. Previous research suggested various potential environmental risk factors for eating disorders, such as dieting and events that trigger mood change and thin-ideal internalization (Chua *et al*., 2004; Striegel-Moore and Bulik, 2007; Haedt-Matt *et al*., 2012). Notably, environmental events shared between siblings might produce unique effects in each individual (Klump *et al*., 2002). For instance, people might have different degrees of thin-ideal internalization under the same social or media exposure. Such differences might be influenced by the age, sex, personality traits and other factors of the individuals, which may also interact with genetic risks (Culbert *et al*., 2015) and need to be considered in risk factor research.

Using extended familial relatedness drastically increased the available sample size of the models in our study, and the use of clinical diagnostic information increased the clinical significance of the results. Our results are consistent with the prior twin study on the overlap between anorexia nervosa and bulimia nervosa based on self-reported measures of eating behaviours (Bulik *et al*., 2010). The consistency confirms the comparability between self-reported eating behavioural measures and clinical measures. Our definition of anorexia nervosa mirrors the definition used in the anorexia nervosa GWAS (Boraska *et al*., 2014; Duncan *et al*., 2017; Watson and Yilmaz, 2019), which also allowed for diagnostic crossover with bulimia nervosa. The largest and most recent GWAS (Watson and Yilmaz, 2019) has reported eight genome-wide-significant genetic polymorphism loci for anorexia nervosa and supported a polygenic aetiology. The genetic correlation found in this study and previous twin research should be revisited using molecular genetic approaches once the GWAS of bulimia nervosa is available.

Limitations of the current study would arise if there are violations of basic assumptions of the models, such as the assumption of equal shared environmental factors across different types of siblings [equal environment assumption, EEA (Plomin *et al*., 2013)] and the assumption of random mating in the population (Nordsletten *et al*., 2016). Previous research has validated EEA in twin studies for multiple psychiatric outcomes (Kendler *et al*., 1993; Conley *et al*., 2013). Although we were unable to validate EEA in full- and maternal half-siblings in our study population, we argue that EEA is as likely to be valid in these two types of sisters as most (91%) children lived with mothers after parental separation (1994). Violation of the random mating assumption threatens the validity of heritability estimation (Plomin *et al*., 2013). Non-random mating has been observed in several psychiatric traits (Frisell *et al*., 2012; Nordsletten *et al*., 2016) and could lead to underestimated heritability in twin studies, as it makes dizygotic twins more genetically similar but does not influence the genetic similarity between monozygotic twins (Plomin *et al*., 2013). However, its influence on heritability estimated from full- and half-sibling data is less predictable. The genetic correlation between two full-sisters might be higher than 0.5 due to non-random mating. However, whether the genetic correlation between two maternal half-sisters in a pair is higher or lower than 0.25 depends on whether their fathers are more or less genetically similar than two random individuals in the population. In addition, the direction and magnitude of bias in heritability estimation due to non-random mating in sibling designs are influenced by how much the genetic correlation between half-sisters deviated from 0.25 in relation to how much the genetic correlation between full-sisters deviated from 0.5. We did not examine the effect of non-random mating on the results, but previous research has suggested that its impact on heritability estimation was mostly mild in twin and sibling studies (Frisell *et al*., 2012; Peyrot *et al*., 2016). Given that the binge-eating and purging type of anorexia nervosa share some core features with bulimia nervosa, distinguishing between different subtypes of anorexia nervosa could potentially provide more insight in understanding the aetiological overlap. However, limited by the available ICD-based diagnostic information in the NPR, we were unable to distinguish the anorexia nervosa subtypes. Nevertheless, the largest GWAS of anorexia nervosa yet conducted (Watson and Yilmaz, 2019) did not find evidence for different polygenic architecture between the restricting and the binge-eating and purging subtypes of anorexia nervosa, although the result should be interpreted carefully in light of low statistical power. Another limitation was that we were only able to detect individuals who were listed in the medical registers. Individuals who had the disorders but were not listed in the registers were therefore misclassified as not having the disorder. Such misclassification might be more common for bulimia nervosa than anorexia nervosa, as we observe a lower than expected prevalence (Smink *et al*., 2012) and bulimia nervosa was not an independent diagnosis in the Swedish version of ICD-9. We were unable to test how such misclassification influenced the results. The issue of misclassification is related to how well a clinical diagnosis reflects the symptom spectrum. A Norwegian twin study showed high genetic correlations between clinical diagnosis and interview-based diagnosis (around 0.8) for major depression and autism spectrum disorder, even though the two methods identified different individuals; however, the genetic correlation between the two measures for alcohol use disorder was lower with wide confidence interval (Torvik *et al*., 2018). Nevertheless, we argue that clinical diagnoses might reflect the more severe end of the spectrum of disordered eating behaviours, and the estimated heritability and genetic correlation might be restricted to the more severe cases.

To our knowledge, this is the first quantitative genetic study on clinically diagnosed anorexia nervosa and bulimia nervosa and their overlap. The moderate and statistically significant genetic and environmental correlations between the two eating disorders might suggest aetiological overlaps and emphasize clinical vigilance of the transition between the two disorders.
